# Multi-omics analysis reveals the crucial role of *ligustrum lucidum* carbon dots in promoting vegetable sweetpotato growth and enhancing soil health

**DOI:** 10.1186/s12870-026-09178-2

**Published:** 2026-06-09

**Authors:** Yiren Su, Zhiyuan Pan, Lukan Zhao, Jiaying Wu, Wenhui Yu, Xibin Dai, Zhilin Zhou, Qinghe Cao

**Affiliations:** 1 Key Laboratory of Sweetpotato Biology and Genetic Breeding, Ministry of Agriculture and Rural Affairs, Xuzhou Institute of Agricultural Sciences in Jiangsu Xuhuai District, Xuzhou, China; 2 School of Life Science, Jiangsu Normal University, Xuzhou, China; 3 Quzhou Academy of Agricultural and Forestry Sciences, Quzhou, China

**Keywords:** Biomass-derived carbon dots, Vegetable sweetpotato, Transcriptomics and metabolomics, Environmental safety, Nanobiostimulant, Sustainable agriculture

## Abstract

**Background:**

The development of sustainable agriculture requires the creation of efficient biostimulants that enhance crop yield and nutritional quality while minimizing environmental impact. Biomass-derived carbon dots, with their unique physicochemical properties and low toxicity, have emerged as promising candidates.

**Results:**

This study synthesized *ligustrum lucidum* carbon dots (LL-CDs) and evaluated their biological effects on vegetable sweetpotato over short-term periods (7 and 14 days). Foliar application of LL-CDs significantly promoted plant growth, biomass accumulation, photosynthetic efficiency, nutrient uptake, and antioxidant capacity. Transcriptomic and metabolomic analyses indicated that LL-CDs treatment synergistically upregulated photosynthesis-related pathways, including light capture, electron transport, and carbon fixation, and increased the accumulation of bioactive compounds. Additionally, LL-CDs improved soil nutrient availability and positively influenced the rhizosphere microbial community structure.

**Conclusions:**

These preliminary results suggest that LL-CDs have the potential to be developed as agricultural nanomaterials, providing initial theoretical support and technical reference for the development of novel biostimulants.

**Supplementary Information:**

The online version contains supplementary material available at 10.1186/s12870-026-09178-2.

## Introduction

With the continuous growth of the global population and the intensification of extreme climate conditions, food security is facing unprecedented challenges. According to an estimate from the FAO’s 2009 report, to meet the global food demand for an expected population of over 9 billion by 2050, global agricultural productivity needs to increase by about 70% [1, 2]. Although precision agriculture and genetic improvement technologies are promising tools for improving crop productivity, their adoption remains constrained by economic, societal, and technological barriers [3]. Currently, the regular application of agrochemicals remains a widely adopted approach to sustaining current levels of agricultural productivity [4]. Nonetheless, prolonged use of conventional agrochemicals may reduce soil biological activity [5], deplete nutrients [6], and diminish biodiversity [7]. Additionally, the presence of pesticide residues in crops poses potential risks to human health [8, 9], such as acute poisoning [10] and chronic diseases [11], ultimately threatening the sustainability of agriculture [12]. In light of these challenges, traditional agriculture shows insufficient adaptability. Therefore, developing novel biofertilizers that can simultaneously enhance yield and ensure ecological sustainability has become a critical issue in agricultural research [13].

Advances in materials science have facilitated the integration of nanotechnology and nanomaterials into agriculture, accelerating the rapid development of modern farming [14]. Studies have shown that nanomaterials have broad applications in agriculture. For example, nanotechnology has made significant progress in the development of eco-friendly pesticides, helping to reduce the use of chemical pesticides [15]; nano-biosensors show promising potential in the early diagnosis and monitoring of crop diseases [16]; furthermore, nanomaterials have been found to enhance plants’ resistance to adverse environmental conditions such as drought and salinity [17]. Despite these benefits, nanomaterials are limited by concerns over their toxicity and poor solubility [18]. Notably, biomass-derived carbon dots demonstrate significant potential in addressing the toxicity issues associated with conventional nanomaterials [19]. This is mainly due to the fact that the carbon precursors of biomass carbon dots come from natural resources (such as plants, agricultural waste, and food residues) [20], which inherently possess biocompatibility. Furthermore, they offer advantages such as ease of surface modification [21], small size [22], and excellent biodegradability [23]. In addition, their green synthesis pathways (hydrothermal methods, microwave-assisted methods) avoid the use of toxic reagents. These approaches are not only cost-effective but also environmentally friendly. Moreover, biomass CDs are rich in surface functional groups such as hydroxyl, carboxyl, and carbon-based moieties. These can act as radical scavengers by donating electrons or protons [24], which activates the plant antioxidant system, eliminates reactive oxygen species (ROS), and mitigates abiotic stress [25]. Their excellent optical properties endow them with great potential in enhancing physiological processes such as photosynthesis, further contributing to increased crop yield [26]. These characteristics confer high chemical reactivity to biomass CDs, making them amenable to further functionalization and expanding their potential applications in agriculture. Xu et al. reported that RR-CDs effectively enhanced photosynthesis in lettuce by increasing chlorophyll content, elevating the net photosynthetic rate, and strengthening the photosystem II electron transport rate [27]. Li et al. demonstrated that foliar application of NCDs promotes assimilate accumulation in rice through upregulating key enzymatic activities in photosynthesis and starch synthesis [28].

Sweetpotato (*Ipomoea batatas* (L.) Lam.) is a globally important food crop with high nutritional value and wide adaptability, playing a crucial role in ensuring food security and promoting dietary diversity [29, 30]. Previous studies have shown that carbon dots (CDs) can influence the stress response mechanisms of traditional storage root sweetpotato through ion signaling pathways mediated by surface functional groups [31, 32]. With the upgrading of consumer demand and adjustments in agricultural industry structure, vegetable sweetpotatoes, which are cultivated for harvesting tender shoots and leaves, have gained increasing attention due to their unique flavor characteristics and high levels of bioactive compounds [33–38]. The yield and nutritional quality of vegetable sweetpotatoes directly determine the economic value of this specialty crop. This study uses *ligustrum lucidum* as the raw material for synthesizing carbon dots, as it is a traditional medicinal herb rich in bioactive components, and it also offers the advantages of being easily accessible and low-cost, making it an ideal sustainable carbon precursor. However, current research on the regulatory mechanisms of *ligustrum lucidum* nanocarbon dots in leaf vegetable sweetpotato is still significantly lacking, especially regarding the effects of carbon dots on photosynthetic carbon metabolism, nutrient distribution, and their interaction with the rhizosphere microenvironment.

Therefore, this study uses the vegetable sweetpotato variety “Xucaishu 7” as the experimental material to systematically investigate the following: (1) the effects of LL-CDs on the growth characteristics, nutritional quality, and key metabolic pathways of leaf vegetable-type sweetpotato; (2) changes in the physico-chemical properties of rhizosphere soil and microbial community structure under short-term LL-CDs treatment; and (3) the physiological and ecological mechanisms by which LL-CDs synergistically enhance the production performance of leaf vegetable-type sweetpotato through the “plant-soil” system. It is hypothesized that after the application of LL-CDs, enzymes related to photosynthesis and nutrient transport proteins in vegetable sweetpotato may be expressed, promoting the accumulation of nutritional components. Short-term (14-day) LL-CDs treatment is expected to improve the rhizosphere soil microenvironment and promote the formation of a beneficial microecological cycle in the root zone, which is favorable for plant growth.

## Materials and methods

### Synthesis and characterization of Carbon Dots (CDs)

3.50 g of aqueous extract from *Ligustrum lucidum* was weighed and dispersed in 70 mL deionized water to obtain a homogeneous suspension. The osmolality was determined using a Fiske Model 210 micro-osmometer (Advanced Instruments, Inc., Norwood, MA, USA). The pH was measured using a pH meter (METTLER TOLEDO-S400, China). The mixture was transferred to a polytetrafluoroethylene (PTFE)-lined stainless-steel autoclave and subjected to hydrothermal treatment at 150 ℃ for 6 h [19]. After cooling to room temperature, the resulting solution was filtered through a 0.22 μm membrane to remove large particulates. The filtrate was further purified using a 3500 Da molecular weight cut-off dialysis membrane and stored at 4 ℃ for subsequent use. The morphology and size distribution of the synthesized CDs were characterized using a transmission electron microscope (TEM; JEM-2100, JEOL, Japan). The surface elemental composition and functional groups were analyzed by X-ray photoelectron spectroscopy (XPS; Thermo Fisher Scientific, USA) and Fourier-transform infrared spectroscopy (FTIR; IRTracer-100, Shimadzu, Japan), respectively. Optical properties were evaluated using a UV–Vis spectrophotometer (UV-2550, Shimadzu, Japan) and a fluorescence spectrophotometer (F-700, Hitachi, Japan), respectively.

### Plant materials

The *Ligustrum lucidum* aqueous extract used in this study was provided by Xuzhou Wegeton Biotechnology Co., Ltd., China, with the product name “*Ligustrum lucidum* Aqueous Extract” and purchased on January 14, 2025. Since this material was commercially acquired and not collected from the wild, no special collection permits were required. The species used is *Ligustrum lucidum* Ait., and the quality inspection was conducted by Xi’an Ruiying Biotechnology Co., Ltd., China. The vegetable sweetpotato variety ‘Xucaishu 7’ was cultivated at the Xuzhou Sweetpotato Research Center (Xuzhou, China; 34°27’N, 117°29’E). Uniform seedlings with three fully expanded leaves and similar growth status were selected. These were transferred to hydroponic containers containing one-quarter strength Hoagland nutrient solution. The plants were maintained at 28 ℃ under a 16 h light / 8 h dark photoperiod with 60% relative humidity.

### Experimental design

Three treatment groups were established: (1) CK: Foliar application of ultrapure water.r (2) LL-H₂O: Foliar application of *Ligustrum lucidum* aqueous extract. (3) LL-CDs: Foliar application of 1.5 mg/mL LL-CDs. Six seedlings were placed in each hydroponic container per group. Each plant received 5 mL of spray solution, ensuring even distribution via fine mist. Treatments were applied every 3 days, with three biological replicates for each group. The nutrient solution was renewed every 3 days.

Soil Experiment: Topsoil (0–20 cm, loam) was collected from a greenhouse at the Sweetpotato Germplasm Resources Research Center (Xuzhou Academy of Agricultural Sciences) using the five-point sampling method. Soil was air-dried, ground, and sieved through a 2 mm mesh. Each pot was filled with 2 kg of prepared soil. The treatment groups included CK and LL-CDs. Twelve seedlings were used in each group. Each plant was sprayed with 5 mL of solution every 3 days. Three biological replicates were included for each treatment. Leaf samples were collected 14 days after treatment for transcriptomic and metabolomic analyses.

### Sample collection

Soil sample collection: Soil samples were collected on days 7 and 14 after treatment, with the collection process divided into two steps: First, the top 2–3 mm of soil at each sampling point was removed to avoid surface contamination. Then, a stainless-steel sampler was used to collect soil at a depth of 15–20 cm. The collected soil samples were immediately stored at -80℃.

Leaf sample collection: Leaf samples were collected on days 7 and 14 after treatment, with uniformly growing plants selected for collection. The collected leaf samples were immediately frozen in liquid nitrogen for 2 min to preserve their physiological state and metabolic characteristics. After freezing, all leaf samples were stored at -80℃.

Rhizosphere soil sample collection: Rhizosphere soil samples were collected on days 7 and 14 after foliar treatment. To analyze the rhizosphere microbial community structure, a sterile brush was used to gently remove the soil adhering to the root surface. Any visible impurities (such as root fragments or large soil particles) were removed, and the soil was then passed through a 20-mesh sieve. The sieved soil was quickly transferred to sterile 1.5 mL microtubes, rapidly frozen in liquid nitrogen, and stored at -80℃.

### Leaf photosynthetic and physiological assessments

Leaf photosynthetic parameters—including net photosynthetic rate (Pn), stomatal conductance (Gs), intercellular CO₂ concentration (Ci), and transpiration rate (Tr)—were measured at 7 and 14 days post-treatment using a portable photosynthesis system (CI-340, CID, USA). Six technical replicates were performed using the second fully expanded leaf from the shoot apex per treatment. On day 14, leaf area and stem length were measured with a vernier caliper. Surface moisture was removed, and the fresh weights of sweetpotato leaves and stems were measured using an analytical balance. Samples were subsequently inactivated at 105℃ for 15 min, then dried at 80℃ to constant weight, and their dry weights were recorded. For physiological analyses, leaves were collected at 7 and 14 days post-treatment, immediately frozen in liquid nitrogen, and stored at − 80℃. Six biological replicates were used per treatment. Activities of superoxide dismutase (SOD), peroxidase (POD), and catalase (CAT), as well as malondialdehyde (MDA) content, were determined using commercial kits (Solarbio, China). Soluble sugar, total carbon, total nitrogen, and total phosphorus contents were measured using commercial assay kits (Solarbio, China) according to the manufacturer’s instructions.

### Soil physicochemical analysis

Rhizosphere soil samples were collected at 7 and 14 days after foliar application of H_2_O or LL-CDs. All soil physicochemical properties were determined using standardized commercial kits and certified reference materials to ensure analytical reproducibility. Soil pH was measured in a 1:2.5 (w/v) soil-to-water suspension after 30 min equilibration with a Mettler Toledo S400 pH meter (Mettler Toledo, Shanghai, China). Soil organic matter (SOM) content was analyzed using a Soil Organic Matter Detection Kit (Cat. No. BC1660, Solarbio Life Sciences, Beijing, China) with a COM-300 A digital titrator (Hiranuma, Tokyo, Japan). Available nitrogen (AN) was determined using a Soil Available Nitrogen Assay Kit (Cat. No. BC1650, Solarbio Life Sciences, Beijing, China). Available potassium (AK) was extracted with 1 M ammonium acetate (pH 7.0) using a Soil Available Potassium Detection Kit (Cat. No. BC1670, Solarbio Life Sciences, Beijing, China) and quantified with a JOYFLY flame photometer (JOYFLY, Shanghai, China). Available phosphorus (AP) was measured using a Soil Available Phosphorus Assay Kit (Cat. No. BC1630, Solarbio Life Sciences, Beijing, China), with absorbance recorded at 880 nm using a DR6000 UV-Vis spectrophotometer (Hach, Loveland, CO, USA). Total potassium (TK) was determined following sodium hydroxide semi-fusion pretreatment using a Soil Total Potassium Detection Kit (Cat. No. BC1680, Solarbio Life Sciences, Beijing, China) and quantified with the same flame photometer. Total phosphorus (TP) was analyzed using a Soil Total Phosphorus Assay Kit (Cat. No. BC1620, Solarbio Life Sciences, Beijing, China), with absorbance detected at 700 nm on the DR6000 spectrophotometer. Total nitrogen (TN) was determined by the Kjeldahl method using a BUCHI K-375 fully automated Kjeldahl analyzer (BUCHI, Flawil, Switzerland), assisted with a Soil Total Nitrogen Assay Kit (Cat. No. BC1610, Solarbio Life Sciences, Beijing, China) for standardized digestion and titration reagents.

### 16S rRNA gene sequencing

Rhizosphere soil samples were collected at 7 and 14 days after foliar application of H_2_O or LL-CDs, with three independent biological replicates per treatment (*n* = 3). Total genomic DNA from soil microbial communities was extracted using the E.Z.N.A.^®^ Soil DNA Kit (Omega Bio-tek, USA) according to the manufacturer’s instructions. DNA concentration was measured using a NanoDrop 2000 spectrophotometer (Thermo Scientific, USA), and integrity was verified by 1% agarose gel electrophoresis. The V3–V4 hypervariable region of the 16S rRNA gene was amplified using barcoded primers 338 F and 806R (Table S1) on a thermal cycler (BIO-RAD, USA). The 20 µL PCR mixture contained 4 µL of 5× TransStart FastPfu buffer, 2 µL of 2.5 mM dNTPs, 0.8 µL of each 5 µM primer, 0.4 µL of TransStart FastPfu DNA polymerase, and 10 ng of template DNA. PCR conditions: initial denaturation at 95℃ for 3 min; 27 cycles of 95℃ for 30 s, 55℃ for 30 s, and 72℃ for 30 s; followed by a final extension at 72℃ for 10 min. Amplicons were stored at 4℃ and subsequently visualized on a 2% agarose gel. Target bands were excised and purified using a DNA Gel Extraction Kit (Yuhua Bio, China). Purified amplicons were used to construct sequencing libraries with the NEXTFLEX Rapid DNA-Seq Kit (PerkinElmer, USA). We compared microbial communities using the Wilcoxon test with FDR correction, and estimated 95% confidence intervals via bootstrap resampling, excluding unclassified data to ensure analysis accuracy.

### Transcriptome sequencing and analysis

Total RNA was extracted from leaves of 3 biological replicates per treatment (CK and LL-CDs, *n* = 3) at 14 days post-treatment. Total RNA was extracted using the FastPure Universal Plant Total RNA Isolation Kit (Vazyme, China). RNA concentration and purity were assessed using a NanoDrop 2000 spectrophotometer (Thermo Scientific, USA) and confirmed by agarose gel electrophoresis. cDNA libraries were constructed by Majorbio (Shanghai, China) using the Illumina^®^ Stranded mRNA Prep, Ligation Kit (Illumina, USA). mRNA was enriched using oligo(dT) beads, fragmented, and reverse transcribed with random hexamers. After end repair and adapter ligation, fragments of 300–400 bp were selected and amplified by 15 cycles of PCR. Libraries were sequenced on the Illumina NovaSeq X Plus platform. Raw reads were processed using fastp for adapter trimming and quality control. Clean reads were aligned to the Ipomoea batatas reference genome (Ipomoea_batatas_pasi3.fa) using HISAT2 (genome source: https://sweetpotato.uga.edu/). Transcript assembly was performed with StringTie, and expression levels were quantified in transcripts per million (TPM) using RSEM. Differentially expressed genes (DEGs) were identified using DESeq2, applying thresholds of |log₂ fold change| ≥ 1 and false discovery rate (FDR) < 0.05. Gene Ontology (GO) and Kyoto Encyclopedia of Genes and Genomes (KEGG) enrichment analyses were conducted using GOATOOLS and SciPy, respectively.

### Metabolite extraction and LC–MS/MS analysis

Freeze-dried leaf samples (50 mg) from 6 independent biological replicates of the CK and LL-CDs treatment groups (*n* = 6) were subjected to metabolite extraction. Metabolomic profiling was conducted by Meiji Biomedical Technology Co., Ltd. (Shanghai, China) using a UHPLC-Q Exactive HF-X system (Thermo Scientific, USA). Metabolite identification and quantification were carried out using Progenesis QI software (Waters, USA), based on MS and MS/MS spectra. Annotations were performed by comparison against the HMDB and Metlin databases. Normalized metabolite data were analyzed using principal component analysis (PCA) and orthogonal partial least squares discriminant analysis (OPLS-DA) with the ‘ropls’ package in R (v1.6.2). Seven-fold cross-validation was used to validate the OPLS-DA model. The screening criteria for differential metabolites were a variable importance in projection (VIP) score > 1.0 and a p-value < 0.05 after false discovery rate (FDR) correction. Significantly altered metabolites were annotated against the KEGG database, and pathway enrichment analysis was conducted using the scipy.stats module in Python with Fisher’s exact test.

### Real-Time Quantitative PCR (qRT-PCR) analysis

To validate the RNA-seq results, quantitative real-time PCR was performed for six selected genes. Specific primers were designed using Primer 5.0 software, and IbACT was used as the internal reference gene (Table S1). Total RNA was extracted using Trizol reagent (Generay, Shanghai, China) according to the manufacturer’s instructions. cDNA synthesis was conducted using the PrimeScript™ RT Reagent Kit (Takara, Japan). qRT-PCR reactions were performed using SYBR Green Realtime PCR Master Mix (TOYOBO, Japan) on a QuantStudio 6 Flex Real-Time PCR System (Thermo Scientific, USA). Relative gene expression levels were calculated using the 2^^−ΔΔCT^ method. All primer sequences used are listed in Table S1.

### Statistical analysis

All data are presented as mean±standard deviation (Mean ± SD). Inter-group differences were analyzed using one-way analysis of variance (One-way ANOVA). If the ANOVA results were significant (*P* < 0.05), post-hoc multiple comparisons were performed using Fisher’s Least Significant Difference (HSD) test. The normality of the data was verified using the Shapiro-Wilk test, and homogeneity of variance was assessed using Levene’s test. If the data did not meet the assumptions of normality or homogeneity of variance, non-parametric tests (Mann-Whitney U test) were used. The significance level was set at *P* < 0.05. Conventional statistical analyses were performed using SPSS Statistics 22 (IBM Corp., USA); bioinformatics analysis of the 16S rRNA sequencing data was conducted based on the QIIME 2 pipeline (version 2021.8). Data visualization was performed using Origin 2019b (OriginLab, USA), and schematic and mechanism diagrams were created using GraphPad Prism 10 and BioRender (https://app.biorender.com/).

## Results

### Characterization of *Ligustrum lucidum* Carbon Dots (LL-CDs)

**Fig. 1:**
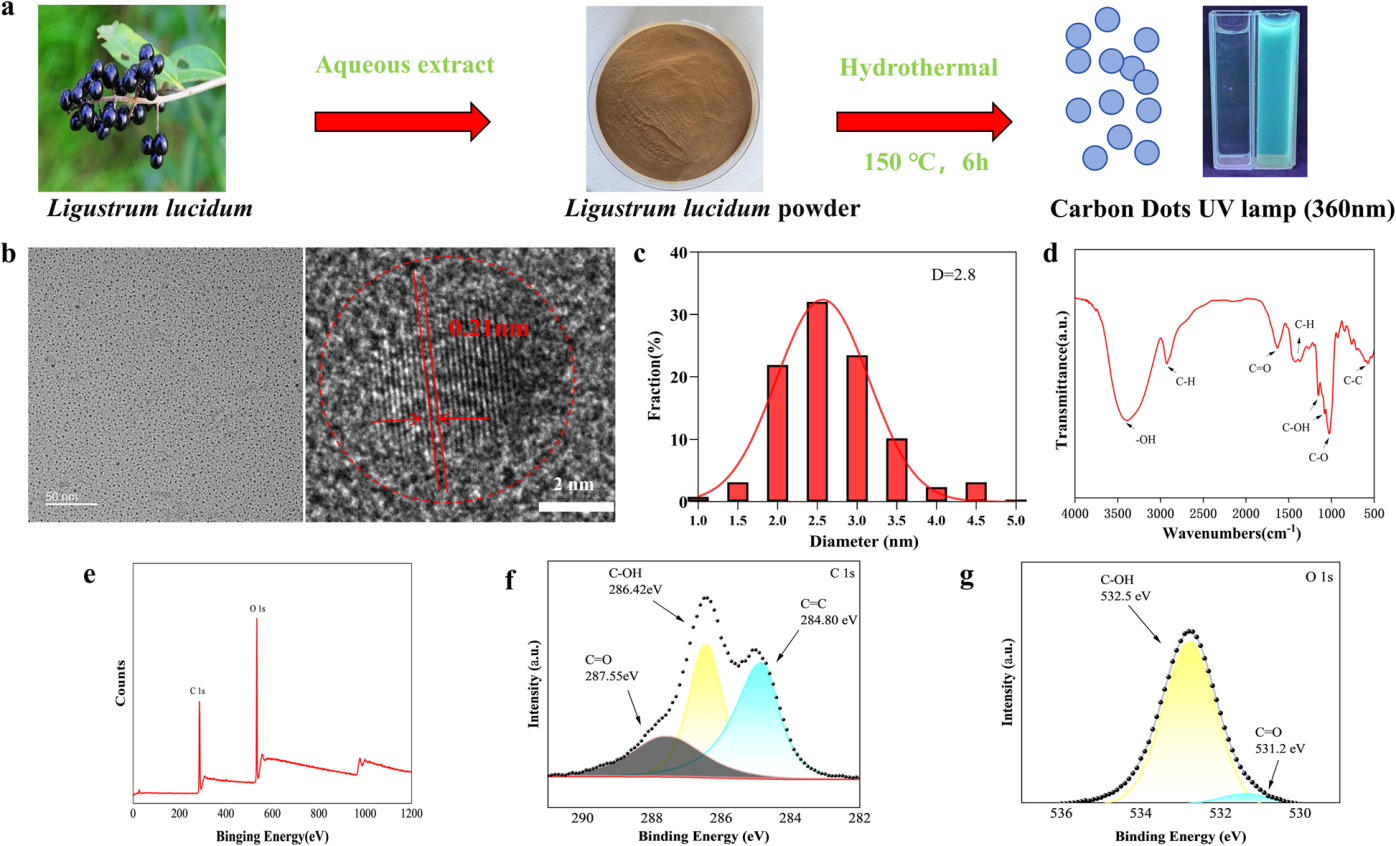
Biosynthesis and characterization of LL-CDs. **a** Images of fresh *Ligustrum lucidum* fruits (left), crude extract (middle), and fluorescence image of the carbon dots (right) under UV light. **b** TEM image of LL-CDs, showing an overall size of 50 nm and individual particle sizes of 2 nm. **c** Size distribution analysis of LL-CDs. **d** Fourier Transform Infrared (FTIR) spectrum of LL-CDs. **e** X-ray Photoelectron Spectroscopy (XPS) survey scan of LL-CDs. **f** High-resolution XPS spectrum of C 1s in LL-CDs. **g** High-resolution XPS spectrum of O 1s in LL-CDs

LL-H_2_O was successfully converted into blue-fluorescent carbon dots (LL-CDs) via a hydrothermal process at 150 ℃ for 6 h (Fig. 1a). The morphology and chemical structure of the resulting LL-CDs were comprehensively characterized using multiple techniques. TEM images revealed that LL-CDs were uniformly dispersed, spherical, and exhibited no noticeable aggregation (Fig. 1b). High-resolution TEM showed clear lattice fringes with an interplanar spacing of 0.21 nm, corresponding to the (100) plane of graphitic carbon (Fig. 1b). Particle size distribution analysis showed an average diameter of 2.8 nm (Fig. 1c). FTIR spectroscopy identified multiple surface functional groups on the LL-CDs, including hydroxyl (–OH), carbonyl (C = O), carboxyl (C–OH), and alkene (C = C) groups (Fig. 1d). The fluorescence quantum yield is 2.1%. XPS further validated the surface chemical composition. The high-resolution C 1s spectrum was deconvoluted into three peaks corresponding to C = C (284.8 eV), C–OH (286.4 eV), and C = O (287.5 eV), indicating the presence of oxygen-containing functional groups on the CD surface (Fig. 1f). Correspondingly, the O 1s spectrum displayed two main peaks at 531.2 eV and 532.5 eV, corresponding to C = O and C–OH bonds, respectively (Fig. 1g). To further explore the origin of the functional groups in LL-CDs, we performed physicochemical characterization of LL-H_2_O and analyzed its components. The pH of LL-H_2_O is 5.7, with an osmotic pressure of 82 mOsm/kg. SEM revealed that the *Ligustrum lucidum* extract exhibited a complex, multiscale architecture (Figure S1). These observations suggest that the extract harbors a diverse array of bioactive compounds, serving as a rich precursor pool for carbon dot synthesis. The results revealed that the broad peak at 3390 cm^− 1^ is attributed to the O-H stretching vibration of hydroxyl groups, a typical feature of polysaccharides and glycosides (e.g., Ligustrum glycosides). The peak at 2926 cm^− 1^ corresponds to the C-H stretching vibration of the methylene group. The absorption peak at 1632 cm^− 1^ is a coupling of O-H bending and C = C stretching vibrations, stemming from the unsaturated components in glycosides. The peaks at 1418 cm^− 1^ and 1369 cm^− 1^ correspond to the C-H shear and bending vibrations of the methylene group. The peaks from 1252 cm^− 1^ to 929 cm^− 1^ are mainly associated with C-O stretching vibrations in polysaccharides and glycosides (pyranose rings, glycosidic bonds, hydroxyl groups), representing key characteristic peaks of polysaccharides. The multiple absorption peaks below 848 cm^− 1^ primarily originate from the vibrations of the pyranose rings in the polysaccharide backbone and C-H out-of-plane wagging vibrations (Figure S2). The results above indicate that the functional groups in LL-CDs mainly originate from LL (*Ligustrum lucidum* aqueous extrac). Additionally, long-term stability assessments revealed that there were no significant changes in the particle size, Zeta potential, or fluorescence intensity of the LL-CDs, confirming their excellent physical stability ( Figure S3).

### LL-CDs promote sweetpotato growth and photosynthetic performance

#### Morphological traits of vegetable sweetpotato

**Fig. 2:**
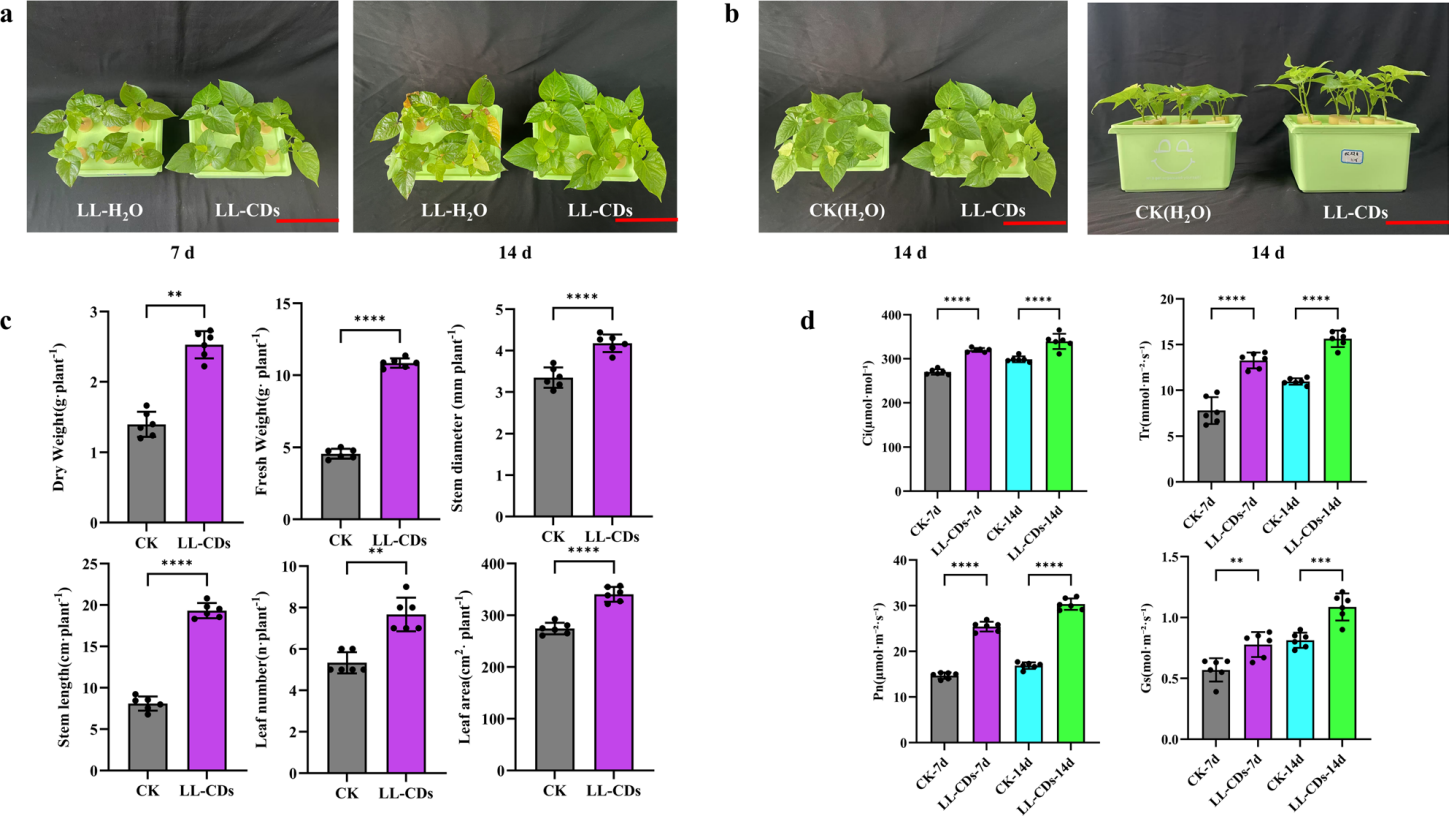
Effects of LL-CDs on growth and photosynthetic performance of vegetable sweetpotato. **a** Representative images of vegetable sweetpotatotreated with LI-H2O and LI-CDs for 7 and 14 days. **b** Growth phenotypes of vegetable sweetpotato under CK (H2O) and LL-CDs treatments after 7 and 14days. **c** Morphological traits measured after 14 days of treatment, including dry weight, fresh weight, stem diameter, stem length, leaf number, and leafarea per plant. **d** Photosynthetic parameters measured on days 7 and 14 under CK and LL-CDs treatments, including net photosynthetic rate (Pn), stomatalconductance (Gs), transpiration rate (Tr), and intercellular CO2 concentration (Ci). Data are presented as mean±standard deviation (*n* = 6). Statistical analysiswas performed using One-way ANOVA followed by Tukey’s HSD test for multiple group comparisons. *p < 0.05, **p < 0.01, ***p < 0.001, ****p < 0.0001;ns, not significant

Foliar application of LL-CDs significantly enhanced the growth of vegetable sweetpotato compared to treatment with LL-H₂O. After 7 days, LL-H₂O-treated plants exhibited mild chlorosis and wilting, along with uneven leaf pigmentation. By day 14, these plants displayed pronounced chlorosis, reduced leaf number, marginal curling, decreased leaf area, and localized blackish-brown lesions on the leaf surface (Fig. 2a), indicating that LL-H₂O exerts inhibitory effects and progressively impairs plant quality. In contrast, LL-CDs-treated plants showed substantial morphological improvements relative to the water control (CK), including broader leaves, significantly elongated stems, uniform pigmentation, and increased leaf density without signs of chlorosis (Fig. 2b). After 14 days of treatment, shoot (leaf + stem) dry weight and fresh weight increased by 80.81% and 137.82% (CK: 4.56 ± 0.34, LL-CDs: 10.85 ± 0.33), respectively. Leaf number, stem length, leaf area, and stem diameter were elevated by 43.75%, 139.01% (CK: 8.09 ± 0.85, LL-CDs: 19.33 ± 0.92), 24.12%, and 24.86%, respectively (Fig. 2c).

#### Photosynthetic parameters

Chlorophyll content and photosynthetic efficiency are key determinants of plant biomass accumulation. In the LL-CDs treatment group, leaf chlorophyll content increased by 79.87% and 109.19% (CK: 1.1 ± 0.08 mg·g^− 1^, LL-CDs: 2.3 ± 0.38 mg·g^− 1^) at 7 and 14 days, respectively, compared to the control. Moreover, LL-CDs treatment significantly improved several photosynthetic parameters—including net photosynthetic rate (Pn), transpiration rate (Tr), intercellular CO₂ concentration (Ci), and stomatal conductance (Gs). Specifically, Pn increased by 73.12% at day 7 and by 79.80% at day 14. Tr exhibited a similar trend, increasing by 70.09% and 42.66% at the respective time points. Ci increased by 18.46% and 13.60%, while Gs rose significantly by 36.55% and 33.61% at days 7 and 14, respectively (Fig. 2d). The concurrent increases in Ci and Gs align with a physiological pattern consistent with stomatal opening. This process may promote CO_2_ assimilation and affect carbon fixation efficiency.

#### Nutritional and stress-related parameters

**Fig. 3:**
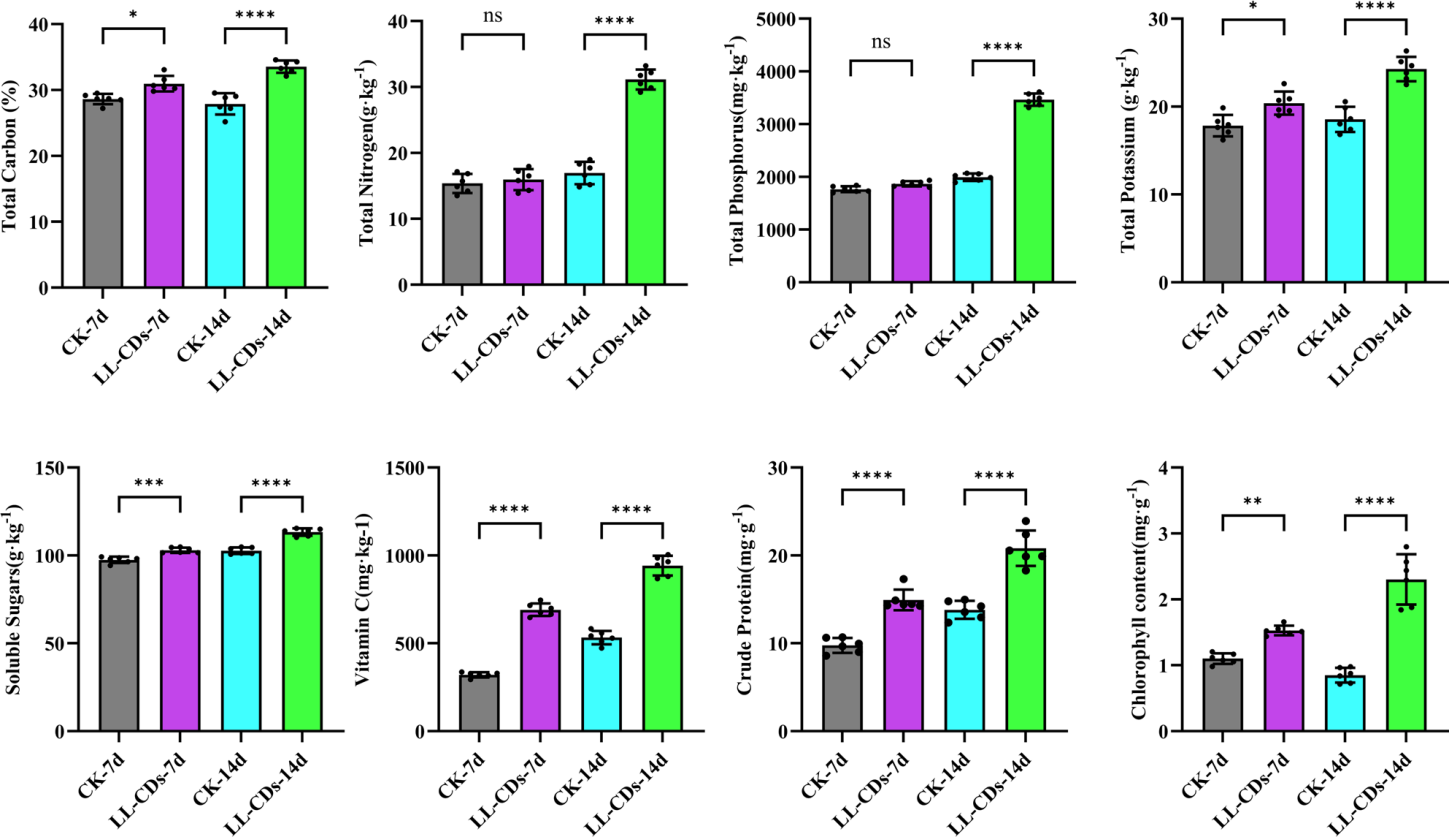
Effects of LL-CDs treatment on nutrient composition in vegetable sweetpotato leaves. **a** Changes in nutrient content of vegetable sweetpotatoleaves after 7 and 14 days of treatment with CK (H2O) and LL-CDs, including total carbon (%), total nitrogen (g·kg− 1), total potassium (g·kg− 1), totalphosphorus (g·kg− 1), soluble sugars (g·kg− 1), chlorophyll content (mg·g− 1), crude protein (mg·g− 1), and vitamin C (mg·kg− 1). Data are presented asmean ± standard deviation (*n* = 6). Statistical analysis was performed using One-way ANOVA followed by Tukey’s HSD test for multiple group comparisons.*p < 0.05, **p < 0.01, ***p < 0.001, ****p < 0.0001; ns, not significant

Macronutrients and antioxidant systems play essential roles in plant growth and development. Therefore, it is important to evaluate the effects of LL-CDs on key physiological parameters, including soluble sugar levels, macronutrient accumulation, and antioxidant enzyme activities in sweetpotato (Fig. 3a). Compared with the control (CK), the soluble sugar content in sweetpotato leaves increased significantly by 5.65% and 10.25% after 7 and 14 days of LL-CDs treatment, respectively (Fig. 3a). In addition, LL-CDs markedly enhanced nutrient element accumulation in leaves over time, especially total carbon and total phosphorus. Specifically, total carbon increased by 8.13% on day 7 and 20.29% on day 14, while total phosphorus increased by 5.84% and 73.72%, respectively. Although no significant increase was observed in total nitrogen and potassium at day 7, both nutrients were significantly elevated by 83.71% and 30.82% at day 14. Antioxidant enzyme assays revealed that CAT activity in LL-CDs-treated leaves significantly increased by 30.48% and 65.94% on day 7 and day 14, respectively. Similarly, SOD and POD activities also exhibited substantial enhancements (65.94%, 48.89%, and 72.61%, 96.62%, respectively). In contrast, the level of MDA, a marker of lipid peroxidation and oxidative stress, significantly declined by 73.80% and 70.86% following LL-CDs application (Figure S4). These findings suggest that LL-CDs not only improve nutrient element content in sweetpotato leaves but also effectively activate the plant’s adaptive antioxidant response, thereby influencing its stress resistance.

#### LL-CDs improve soil nutrient status

Eight soil indicators were assessed to evaluate rhizosphere nutrient status and overall soil health. Analysis of physicochemical parameters and nutrient levels revealed that LL-CDs treatment significantly improved the nutrient environment and physicochemical characteristics of the rhizosphere soil of sweetpotato. Regarding nitrogen availability, LL-CDs treatment markedly increased both total nitrogen (TN) and available nitrogen (AN) contents. Notably, on day 14, total nitrogen content was 34.55% higher than the corresponding control, and the enhancement in available nitrogen was particularly prominent (23.53%) (Figure S5), indicating that LL-CDs enhance nitrogen availability and uptake efficiency. Potassium availability was also markedly improved by LL-CDs application. Both available potassium (AK) and total potassium (TK) levels were consistently higher in the LL-CDs-treated group at both 7 and 14 days, with the most pronounced increases observed on day 7 (7.33% and 22.97%, respectively) (Figure S5). In contrast, only available phosphorus (AP) showed a significant change in the treatment on day 14, while total phosphorus (TP) remained stable. Additionally, a slight decrease in soil pH was observed under LL-CDs treatment, although pH remained within the normal range. This mild acidification may contribute to alleviating alkaline soil stress. Moreover, organic matter (OM) content increased significantly by 11.84% at day 14, further supporting the role of LL-CDs in improving soil fertility (Figure S5). In summary, LL-CDs application enhanced the nitrogen and potassium supply capacity, modulated soil pH, and promoted the accumulation of organic matter. These changes collectively optimized the soil’s nutrient profile and physicochemical properties, providing a more favorable environment for healthy sweetpotato growth in the short term.

#### 16S rRNA gene sequencing

**Fig. 4:**
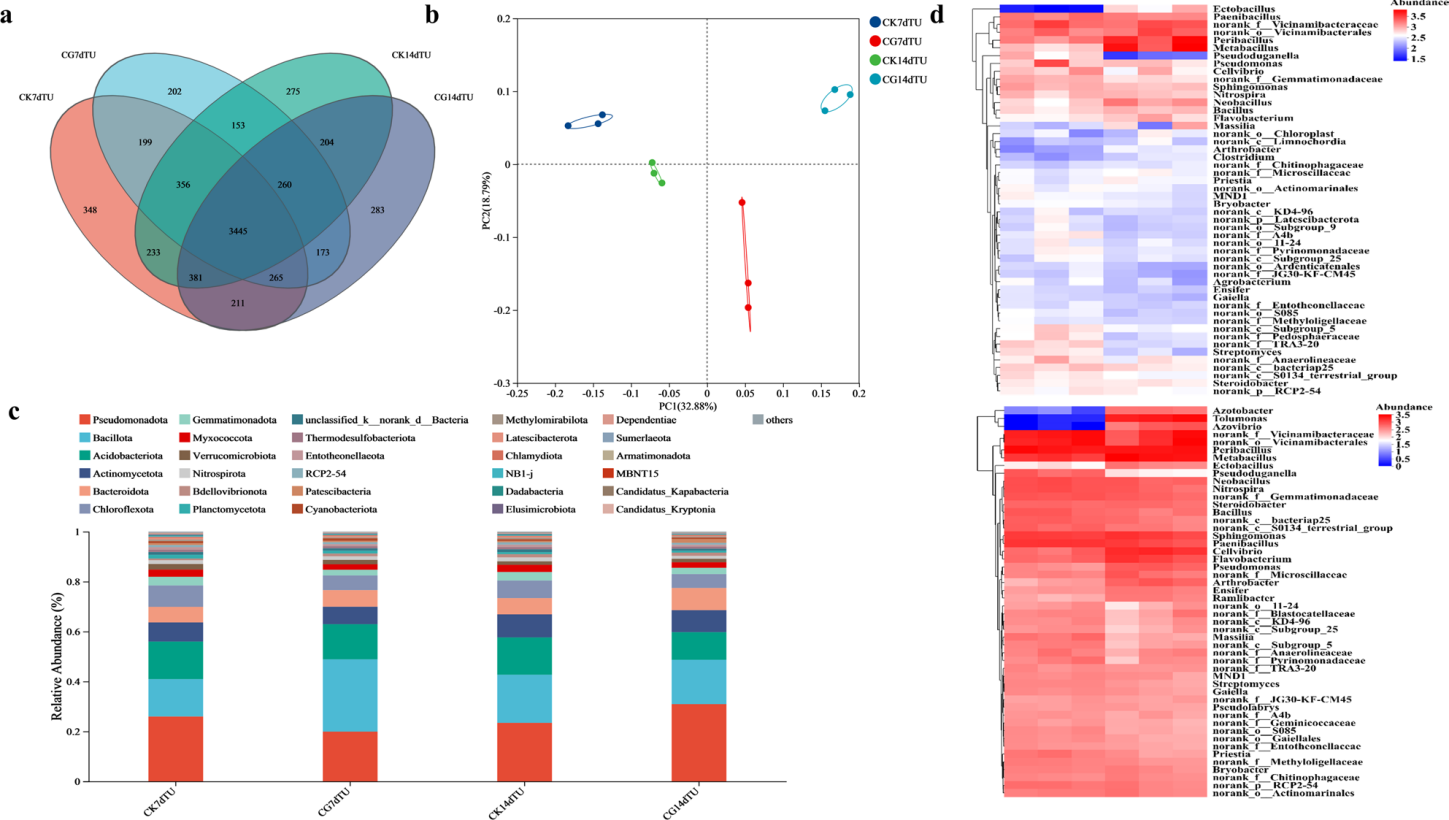
Effects of LL-CDs treatment on rhizosphere microbial community structure. **a** Venn diagram of soil microbial OTUs among the four treatment groups: CK7dTU, LL-CDs7dTU, CK14dTU, and LL-CDs14dTU. **b** Principal coordinate analysis (PCoA) of microbial communities after 7 and 14 days of treatment. **c** Taxonomic composition and relative abundance of bacterial communities at the phylum level across different treatments. Data represent the average of biological replicates. **d** Heatmaps showing the relative abundance of dominant bacterial genera under different treatments at 7 and 14 days. Color gradients represent Z-score normalized abundance values

To investigate the ecological safety of LL-CDs on soil microbial diversity, 16S rRNA gene sequencing was performed (biological replicates *n* = 3). Venn diagram analysis revealed that 3,465 core operational taxonomic units (OTUs) were shared among the four groups (CK7dTU, CG7dTU, CK14dTU, and CG14dTU), with each group also harboring distinct OTUs. Notably, 202 and 283 unique OTUs were identified in the CG7dTU and CG14dTU groups, respectively, indicating time- and treatment-specific enrichment of microbial taxa (Fig. 4a). Principal coordinates analysis (PCoA) showed distinct clustering of LL-CDs-treated versus control groups along the PC1 axis, which accounted for 32.88% of total variation (Fig. 4b), indicating that LL-CDs significantly influenced bacterial community composition. At the phylum level, bacterial communities across all treatments were predominantly composed of *Pseudomonadota*, *Actinobacteriota*, *Acidobacteriota*, *Chloroflexi*, and *Bacteroidota* (Fig. 4c). In particular, the relative abundances of *Pseudomonadota* and *Bacteroidota* were markedly elevated in the CG14dTU group compared with CK14dTU, implying that LL-CDs may promote the proliferation of phyla involved in nutrient cycling and stress responses. Further clustering analysis (Fig. 4d) revealed distinct differences in microbial composition between the treatment and control groups. Genera such as *Azotobacter*, *Tolumonas*, and members of the family *Vicinamibacteraceae* were significantly enriched in the CG14dTU group but were nearly undetectable in CK14dTU, suggesting their enhanced functional roles in nitrogen fixation and carbon cycling. Additionally, dominant genera such as *Flavobacterium* and *Sphingomonas* were present in both groups but showed increased relative abundance under LL-CDs treatment, indicating a shift toward a more functionally diverse and metabolically active microbial ecosystem. Overall, these results suggest that short-term application of LL-CDs may selectively enriched beneficial bacterial taxa with potential roles in organic matter degradation, nutrient transformation, and plant growth promotion. However, their long-term ecological effects still require further evaluation through sustained research.

### Transcriptomic analysis

**Fig. 5:**
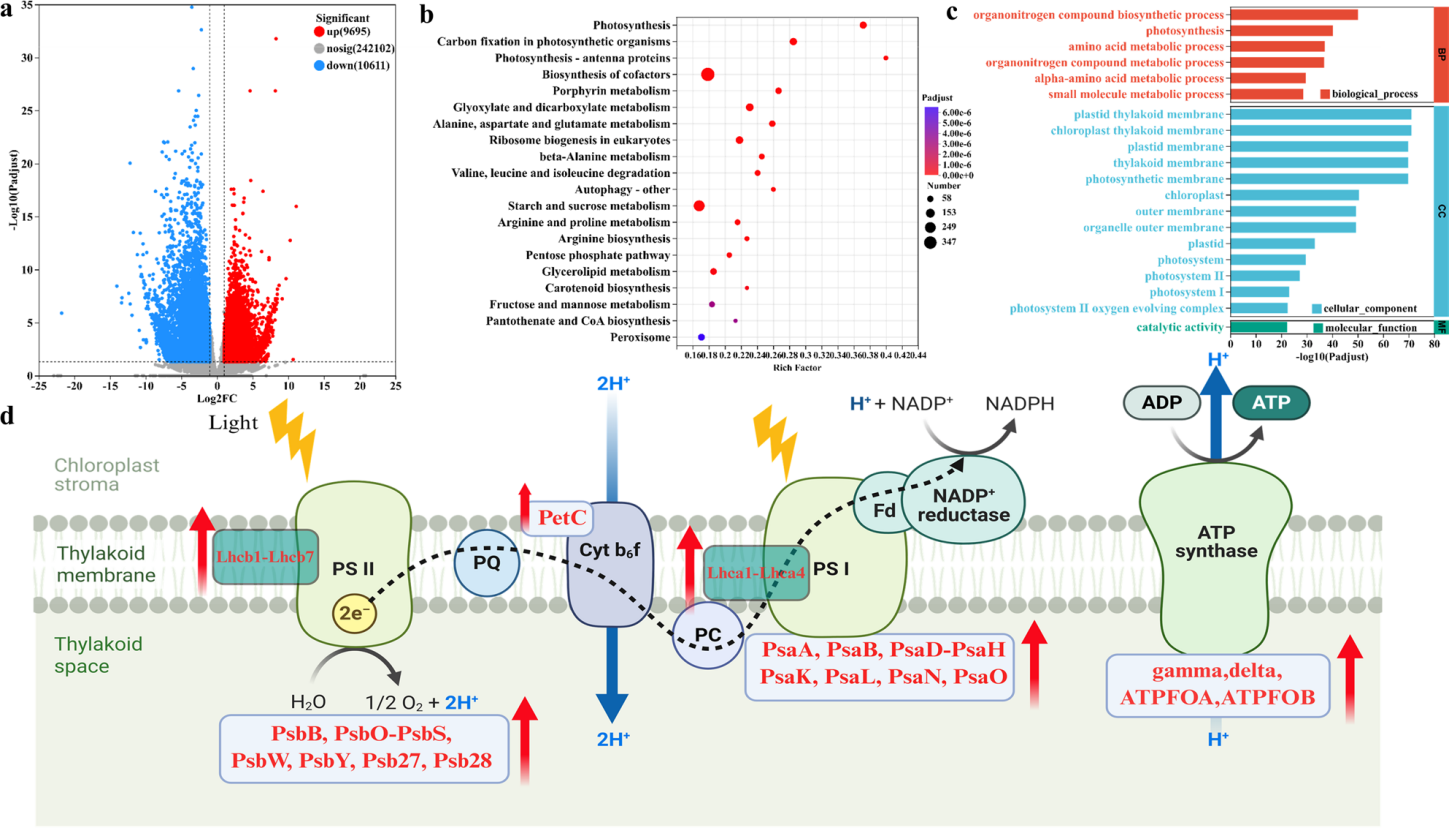
Global statistical overview and functional annotation of Differentially Expressed Genes (DEGs). **a** Volcano plot of DEGs between CKY and CLY groups. Red dots represent significantly up-regulated genes, blue dots represent significantly down-regulated genes, and grey dots indicate non-significant genes (|log₂FC| > 1, *p* < 0.05). **b** Gene Ontology (GO) enrichment analysis of DEGs categorized into Biological Process (BP), Cellular Component (CC), and Molecular Function (MF). The x-axis indicates the significance level as −log_10_(p-adjust). **c** KEGG pathway enrichment of DEGs. The y-axis represents enriched pathways, the x-axis shows the rich factor (ratio of DEGs to total genes in the pathway), bubble size indicates the number of genes, and bubble color reflects the adjusted p-value (Padj). **d** Thylakoid electron transport chain showing upregulated genes under LL-CDs treatment. Red arrows indicate significantly upregulated genes in PSII, cytochrome b6f, PSI, ATP synthase, and light-harvesting complexes

To elucidate the molecular mechanisms underlying the effects of LL-CDs on vegetable sweetpotato, six cDNA libraries were constructed from aerial tissues (leaves and stems) for transcriptome sequencing (biological replicates *n* = 3). A total of 42.63 Gb of high-quality data was obtained, with an average of 6.62 Gb per library. The Q30 scores of all libraries exceeded 95.89%, with GC content ranging from 44.28% to 45.10%. The average error rate, Q20, and Q30 were 0.012%, 98.70%, and 96.96%, respectively. Total read mapping rates exceeded 91.71% for all samples, confirming the suitability of the reference genome. Among the clean reads, 82.39% aligned to exonic regions, 5.04% to intronic regions, and 3.35% to intergenic regions (Table S2), indicating high transcriptomic coverage and sequencing quality. Principal component analysis (PCA) showed that PC1 and PC2 explained 63.22% and 12.40% of the total gene expression variation, respectively, indicating substantial transcriptomic differences between the LL-CDs-treated and control (CK) groups (Figure S6). Correlation heatmap analysis further confirmed the high consistency among biological replicates (Figure S7). A total of 20,306 differentially expressed genes (DEGs) were identified, including 9,695 upregulated and 10,611 downregulated genes (Fig. 5a). The large number of DEGs may be attributed to the high gene redundancy in sweetpotato as a heterozygous hexaploid crop. Thus, the identified DEGs may include functionally similar gene family members, reflecting the complex gene expression changes in polyploid plants in response to environmental stimuli. Functional classification of DEGs (Figure S8) revealed enrichment in several key biological processes, including posttranslational modification, protein turnover, chaperones, transcription, signal transduction mechanisms, and carbohydrate transport and metabolism, involving 1,427, 1,190, 1,310, and 973 genes, respectively. To elucidate the biological significance of the DEGs, GO enrichment analysis (Fig. 5b) showed significant enrichment in biological processes (e.g., photosynthesis, organic nitrogen compound biosynthesis), cellular components (e.g., thylakoid and plastid membranes), and molecular functions (e.g., catalytic activity). KEGG pathway analysis (Fig. 5c) revealed that DEGs were significantly involved in photosynthesis, carbon fixation, porphyrin metabolism, cofactor biosynthesis, and amino acid metabolism, particularly the alanine, aspartate, and glutamate pathways. Validation of gene expression was performed and is presented in the Figure S9.

### Transcriptional enhancement of photosynthesis

Transcriptome profiling revealed a coordinated transcriptional upregulation across the entire photosynthetic pathway, encompassing antenna protein assembly, chlorophyll biosynthesis, photosynthetic electron transport, and carbon fixation (Fig. 5d). Notably, genes encoding light-harvesting complex proteins (Lhca1-4, Lhcb1-7; ko00196) were significantly induced, which is consistent with enhanced light-capturing capacity (Figure S10). Concurrently, chlorophyll biosynthesis genes (e.g., HemB, HemE, CHLG; ko00860) (Figure S11) and key enzymes in the photosynthetic electron transport chain (e.g., PsbB, PsaA, PetC, ATPFOA; ko00195) (Figure S12) showed elevated expression, suggesting that photochemical energy conversion may be strengthened. This upstream enhancement was accompanied by increased expression of Calvin-Benson cycle enzymes (PRK, SBPase, PGK; ko00710), reflecting augmented CO_2_ assimilation (Figure S13). Collectively, these transcriptional changes depict a stepwise reinforcement of photosynthetic capacity from light harvesting to carbon fixation (Fig. 5d).

### Metabolomic analysis

**Fig. 6:**
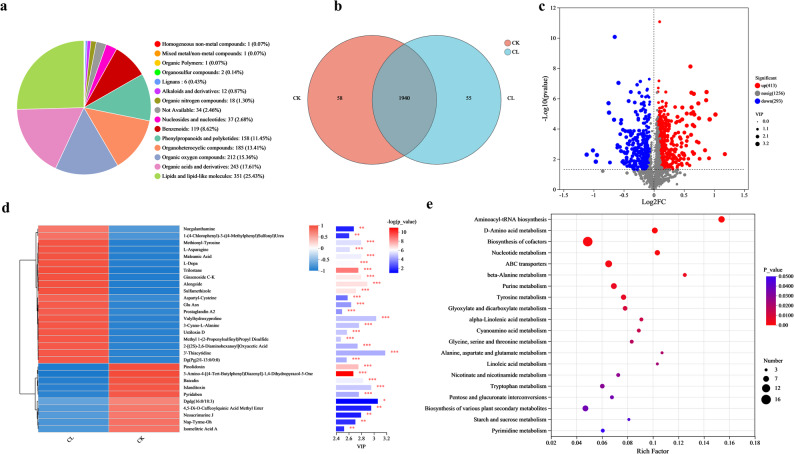
Metabolomic profiling and pathway enrichment analysis between control (CK) and treatment (DW) groups. **a** Classification of identified metabolites based on chemical taxonomy. **b** Venn diagram showing the overlap of detected metabolites between the CK and DW groups. **c** Volcano plot of Differentially Accumulated Metabolites (DAMs) between CK and DW. Red and blue dots represent significantly up-regulated and down-regulated metabolites, respectively (VIP > 1, |log₂FC| > 1, *p* < 0.05); gray dots represent non-significant differences. **d** Heatmap of key DAMs with hierarchical clustering (left) and corresponding VIP scores and significance values (right). **e** KEGG pathway enrichment analysis of DAMs. The y-axis lists enriched pathways; the x-axis shows the rich factor (ratio of DAMs to total compounds in the pathway); bubble size indicates the number of enriched metabolites, and color represents *p*-value

To investigate the global metabolic alterations induced by LL-CDs in sweetpotato leaves, an untargeted metabolomic profiling was conducted (biological replicates *n* = 6). This analysis resulted in the identification of 2,064 metabolites. These metabolites were classified into 15 distinct categories (Fig. 6a), with lipids and lipid-like molecules, organic acids and derivatives, organic oxygen compounds, organoheterocyclic compounds, and phenylpropanoids and polyketides constituting the major groups. This compositional profile reflects a broad spectrum of functional responses associated with membrane structure, energy metabolism, antioxidative defense, signal transduction, and stress adaptation under LL-CDs treatment. PCA revealed clear separation between the control (CK) and the LL-CDs-treated (CL), with PC1 and PC2 accounting for 55.50% and 19.60% of the total variance, respectively (Figure S14). A total of 58 metabolites were unique to the CK group and 55 to the CL group (Fig. 6b). Comparative analysis identified 706 differentially expressed metabolites (DEMs), including 413 upregulated and 293 downregulated in the CL group relative to CK (Fig. 6c). Hierarchical clustering and variable importance in projection (VIP) analyses further confirmed the distinct metabolic profiles between the two groups (Fig. 6d). Notably, metabolites(Level 2 annotation confidence) such as norgalanthamine, methionyl-tyrosine and L-DOPA were significantly enriched in the CL group. To explore the underlying metabolic mechanisms, the DEMs were subjected to KEGG pathway enrichment analysis (Fig. 6e). The most significantly enriched pathways included aminoacyl-tRNA biosynthesis, D-amino acid metabolism, and cofactor biosynthesis. Several amino acid metabolic pathways, including tyrosine metabolism, alanine, aspartate and glutamate metabolism, as well as nucleotide metabolism were also significantly enriched. ABC transporters and starch and sucrose metabolism pathways were among the enriched terms. In parallel, the biosynthesis of various plant secondary metabolites pathway was significantly enriched, accompanied by the upregulation of multiple secondary metabolites (Table S3). Collectively, these results demonstrate that LL-CDs treatment induces extensive changes in the sweetpotato leaf metabolome, with significant modulations observed in multiple primary and secondary metabolic pathways.

### Co-analysis of DEGs and DAMs

**Fig. 7:**
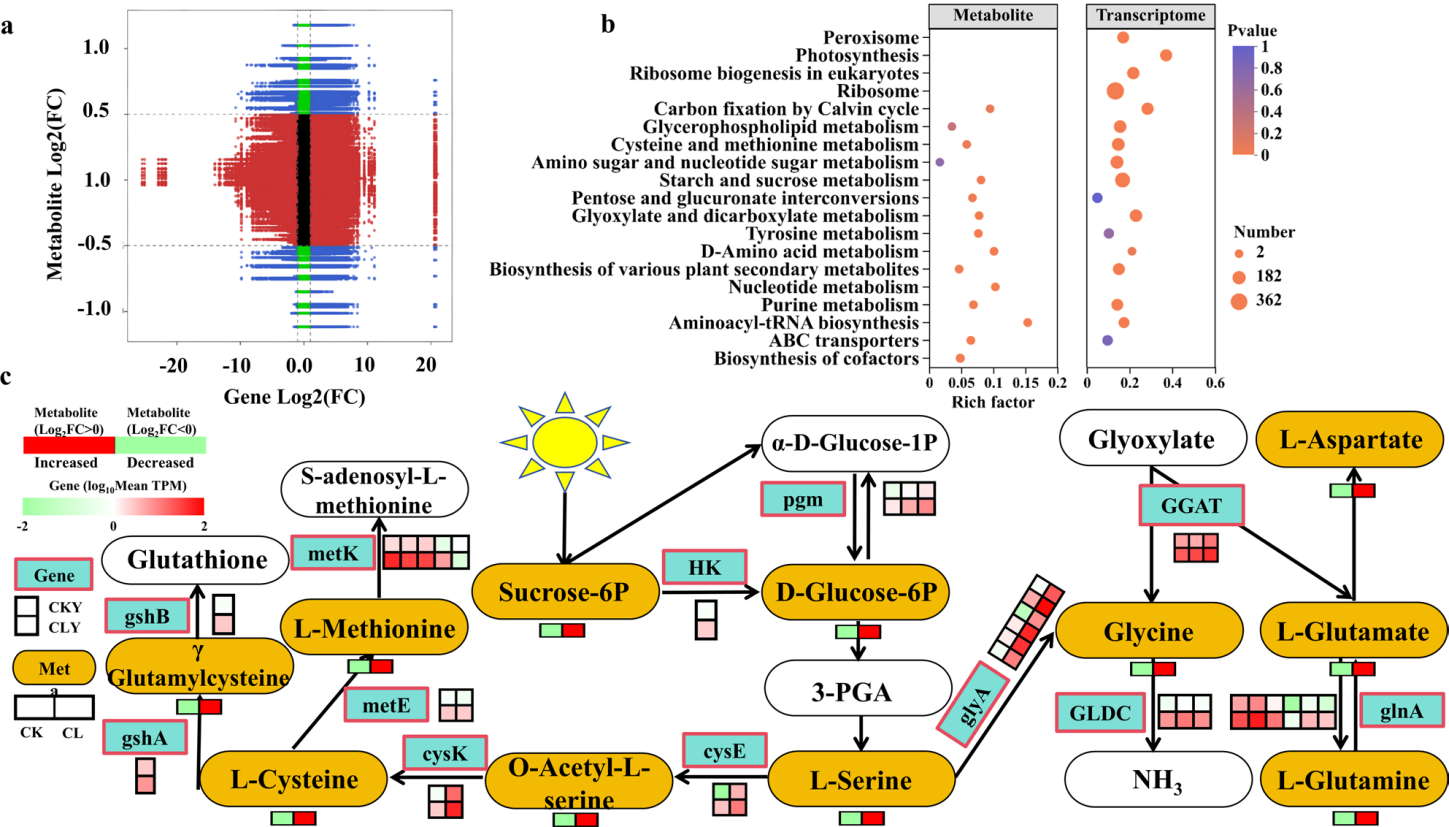
Integrated transcriptomic and metabolomic analysis and alterations in major metabolic pathways. **a** Nine-quadrant plot showing the correlation between DEGs and DAMs in the CL vs. CK comparison. **b** KEGG enrichment analysis of transcriptomic (right) and metabolomic (left) datasets. Bubble size indicates the number of enriched features; color represents p-value; the x-axis shows the rich factor. **c** Representative pathways include cysteine and methionine metabolism, starch and sucrose metabolism, alanine, aspartate and glutamate metabolism, and glyoxylate and dicarboxylate metabolism. Upper rectangles indicate DEGs; lower rectangles indicate DAMs. Red and green denote upregulation and downregulation, respectively. Yellow ellipses represent significantly changed metabolites, white ellipses indicate undetected or non-significant metabolites. Blue boxes mark enzymes affected by DEGs. Each rectangle is divided into two parts representing DAMs (CK vs. CL) and DEGs (CKY vs. CLY); the color reflects the proportion of regulation under LL-CDs treatment as shown in the scale. CK and CKY: control groups sprayed with H₂O; CL and CLY: treatment groups sprayed with LL-CDs

To clarify the relationship between DEGs and DAMs under LL-CDs treatment, a correlation-based integration analysis was performed. This analysis applied a co-expression network approach (Pearson correlation coefficient > 0.8, *p* < 0.05). The results showed that most data points clustered in the third and seventh quadrants (Fig. 7a), reflecting a positive association between gene expression levels and corresponding metabolite accumulation. KEGG enrichment analysis revealed that both the transcriptomic and metabolomic datasets shared enrichment in 12 key metabolic pathways (Fig. 7b). This overlap suggests a potential coordinated regulation between the two omics layers. Notably, alanine–aspartate–glutamate metabolism and glycine–serine–threonine metabolism pathways were co-enriched. The activation of these pathways was observed alongside the accumulation of plant biomass. Furthermore, in the CL group, where biomass increased significantly, key nitrogen metabolites (glutamate and aspartate) accumulated, and the starch and sucrose metabolism pathways were also enriched. The concurrent enrichment of secondary metabolite biosynthesis pathways suggests that the production of bioactive compounds may have been maintained during growth under LL-CDs treatment. Importantly, several genes involved in carbon, nitrogen, and sulfur metabolism—including HK, pgm, cysE, cysK, gshA, gshB, metE, metK, glyA, GGAT, GLDC, and glnA—were significantly upregulated under LL-CDs treatment. In parallel, related metabolites such as sucrose-6-phosphate, D-glucose-6-phosphate, L-serine, L-cysteine, γ-glutamylcysteine, glutathione, L-methionine, glycine, and glutamate also showed significant increases (Fig. 7c). The schematic diagram of the joint analysis is shown in Figure S15.

## Discussion

Vegetable sweetpotato is renowned for its exceptional nutritional and functional value, offering both economic and health benefits [39–41]. However, its large-scale production is constrained by yield limitations, and the use of conventional agrochemicals raises concerns regarding ecological safety [42, 43]. Therefore, the development of efficient, low-toxicity, and environmentally friendly biostimulants is crucial. Biomass-derived carbon dots (CDs) exhibit good biocompatibility and substantial application potential [44]. In this study, we systematically elucidated the growth-promoting and secondary metabolism-enhancing effects of LL-CDs on vegetable sweetpotato from both physiological and molecular perspectives, and confirmed their short-term ecological safety, providing a novel strategy for green agriculture.

Our results demonstrate that foliar application of LL-CDs significantly promoted the growth of sweetpotato, as evidenced by increased aboveground biomass and improved morphological features of leaves, stems, and branches. LL-CDs treatment also enhanced gas exchange parameters and nutrient accumulation, indicating improved nutrient absorption and transport. Characterization of LL-CDs revealed uniform particle size (2.8 nm) and a graphitic nanostructure with surface oxygen functional groups. These functional groups facilitate cellular penetration, enhance bioavailability, and promote nutrient absorption [45–48]. Additionally, LL-CDs can chelate metal ions and increase the ability to scavenge free radicals [49], thereby mitigating oxidative stress [50]. Furthermore, the excellent colloidal stability of LL-CDs ensures uniform coverage on the leaf surface, facilitating sustained absorption [51]. In contrast, the complex macromolecules in LL-H_2_O tend to precipitate, which may negatively affect stomatal function. These properties collectively contributed to enhanced plant growth.

After 14 days of LL-CDs treatment, the carbon, nitrogen, phosphorus, and potassium contents in the plants were significantly higher than those in the control group, likely due to the assistance of oxygen functional groups in membrane transport of nutrient ions [52]. Moreover, the stable negative potential (-7.5 to -8.0 mV) facilitated the formation of a stable colloidal system, further enhancing nutrient absorption [53]. LL-CDs also promoted the accumulation of bioactive metabolites, including soluble sugars, proteins, chlorophyll, and vitamin C. The increase in vitamin C (a non-enzyme antioxidant), along with changes in the activities of peroxidase (POD), catalase (CAT), superoxide dismutase (SOD), and the content of malondialdehyde (MDA), indicates that LL-CDs likely activated the plant’s antioxidant system, alleviating oxidative stress. This mechanism may be related to the high photostability and graphite structure of LL-CDs, which help maintain thylakoid membrane stability [54, 55], reduce photoinhibition, and improve physiological adaptability under light stress.

Transcriptomic and metabolomic analyses revealed upregulation of the genes related to light-harvesting complexes and key enzymes involved in chlorophyll biosynthesis, suggesting enhanced photosynthetic efficiency [56–59]. This may further improve electron transport, increase ATP/NADPH production, and enhance carbon fixation [60–63], thereby promoting CO_2_ assimilation and RuBP regeneration [64, 65]. Additionally, the upregulation of amino acid synthetase expression stimulated nitrogen and sulfur metabolism, potentially enhancing protein synthesis and oxidative stress tolerance [66–68]. Overall, we hypothesize that LL-CDs may impact crop light capture and pigment synthesis, enhance photochemical reactions, drive carbon fixation, and synergistically activate carbon-nitrogen-sulfur integrated metabolism, potentially promoting biomass accumulation and metabolic efficiency.

Assessing the ecological safety of novel plant-regulating nanomaterials is a key factor for ensuring agricultural sustainability. Our findings show that LL-CDs treatment increased the availability of soil nutrients, particularly total nitrogen, available nitrogen, and potassium. These changes provided a more nutrient-rich environment for plant growth. At the microbial community level, 16S rRNA gene sequencing indicated that LL-CDs treatment increased the relative abundance of beneficial microorganisms such as Pseudomonas and Bacillus, which are associated with nutrient cycling, nitrogen fixation, and plant growth promotion [69–74]. However, these changes did not reach statistical significance, indicating that the effects of LL-CDs on microbial composition are relatively mild. Given the short experimental period of 14 days, further long-term studies are needed to confirm the impact of LL-CDs on soil microecology (Figure S16).

## Conclusion

This study systematically explored the integrated biological effects of LL-CDs in vegetable sweetpotato, including growth promotion, metabolic regulation, and environmental safety. The results indicated that LL-CDs promoted photosynthetic efficiency, improved nutrient utilization, and ultimately enhanced biomass accumulation. LL-CDs influenced key pathways such as chlorophyll biosynthesis, antioxidant defense, and carbon-nitrogen-sulfur metabolism, directly forming a transcriptionally regulated network that supports nutrient coordination and redistribution. These mechanisms collectively promoted plant growth and development. Environmental assessments showed that short-term foliar application of LL-CDs had no adverse effects on soil physicochemical properties or rhizosphere microbial communities, indicating good ecological compatibility. Overall, LL-CDs are a green nanomaterial with excellent biological functionality and sustainability, demonstrating broad application potential.

## Supplementary Information

Supplementary Material 1.

supplementary materials

## Data Availability

Raw datasets generated from RNA-seq and 16S rRNA sequencing are accessible in the NCBI SRA database under accession numbers SUB15449436 and SUB15465817, respectively. Metabolomics data are available in the MetaboLights database under accession number REQ20250711211728. The datasets supporting the conclusions of this article are included within the article (and its additional files).

## Abbreviations

CDs: Biomass-derived carbon dots
GO: Gene Ontology
LL-CDs: *Ligustrum lucidum* Carbon Dots
BP: Biological Process
LL: *Ligustrum lucidum* aqueous extrac
MF: Molecular Function
CC: Cellular Component
ROS: Reactive Oxygen Species
KEGG: Kyoto Encyclopedia of Genes and Genomes
PTFE: Polytetrafluoroethylene
qRT-PCR: Real-Time Quantitative PCR
TEM: Transmission electron microscope
SD: Standard deviation
XPS: X-ray photoelectron spectroscopy
HSD: Honestly Significant Difference
FTIR: Fourier-transform infrared spectroscopy
TN: Total nitrogen
SEM: Scanning electron microscopy
AN: Available nitrogen
LL-H_2_O: Aqueous extract of *Ligustrum lucidum*
AK: Available potassium
Pn: Photosynthetic rate
TK: Total potassium
Gs: Stomatal conductance
TP: Total phosphorus
Ci: Intercellular CO_2_ concentration
AP: Available phosphorus
Tr: Transpiration rate
PCoA: Principal coordinates analysis
SOD: Superoxide dismutase
CK: Control group
POD: Peroxidase
CL: LL-CDs-treated group
CAT: Catalase
DEMs: Differentially expressed metabolites
MDA: Malondialdehyde
VIP: Variable importance in projection
DEGs: Differentially expressed genes
OUTs: Operational taxonomic units
