# Supplementary material for: Multi-omics analysis reveals the crucial role of ligustrum lucidum carbon dots in promoting vegetable sweetpotato growth and enhancing soil health
Source: BMC Plant Biol. 2026 Jun 9;26:1387. doi: 10.1186/s12870-026-09178-2 (PMC13474892; doi:10.1186/s12870-026-09178-2)
Supplement: Supplementary file 2 [file 12870_2026_9178_MOESM1_ESM.docx]

**Supplementary Material**

**Fig. S1**. Scanning electron microscopy (SEM) images of LI (*Ligustrum lucidum* aqueous extrac)

**Fig. S2**. **Infrared spectral characteristics of *Ligustrum lucidum* crude extract samples**

**Fig. S3**. Temporal changes in hydrodynamic size (a), zeta potential (b), and fluorescence intensity (c) of LI-CDs (n = 3), with comparative FTIR spectra of LI-CDs and LI (d). Green-shaded regions represent identical absorption peaks, and red-shaded regions highlight differential absorption peaks.

**Fig. S4**. Antioxidant enzyme activities and malondialdehyde (MDA) content in sweetpotato leaves. Peroxidase (POD), catalase (CAT), and superoxide dismutase (SOD) activities are expressed as U·mg⁻¹ protein, and MDA content is expressed as nmol·mg⁻¹ protein. Data are presented as mean ± standard deviation (n = 6). Statistical analysis was performed using One-way ANOVA followed by Tukey's HSD test for multiple group comparisons. *p < 0.05, **p < 0.01, ***p < 0.001, ****p < 0.0001; ns, not significant.

**Fig. S5. Effects of LI-CDs treatment on soil physicochemical properties.**

Changes in major soil physicochemical properties after 7 and 14 days of treatment with CK (H₂O) and LI-CDs, including total nitrogen (g·kg⁻¹), total phosphorus (g·kg⁻¹), available phosphorus (mg·kg⁻¹), alkali-hydrolyzable nitrogen (mg·kg⁻¹), available potassium (mg·kg⁻¹), total potassium (g·kg⁻¹), pH, and organic matter content (g·kg⁻¹). Data are presented as mean ± standard deviation (n = 6). Statistical analysis was performed using One-way ANOVA followed by Tukey's HSD test for multiple group comparisons. *p < 0.05, **p < 0.01, ***p < 0.001, ****p < 0.0001; ns, not significant.

**Fig. S6**. Principal component analysis (PCA) of RNA-seq samples. PCA was performed based on the normalized gene expression profiles of all samples. Triangles represent the CLY group and circles represent the CKY group.

**Fig. S7**. Correlation analysis of RNA-seq expression profiles among all samples. Pearson correlation coefficients were calculated based on normalized gene expression levels across all biological replicates. The color scale indicates the strength of correlation (from blue = low to red = high), and the size of the circles represents the magnitude of correlation coefficients.

**Fig. S8**. COG functional classification of differentially expressed genes (DEGs). The DEGs were functionally categorized based on Clusters of Orthologous Groups (COG) annotations. The X-axis represents functional categories (A–Z), and the Y-axis indicates the number of genes assigned to each category.

**Fig. S9**. Validation of gene expression by qRT-PCR.. The expression levels of six representative genes were validated in CL and CK samples using quantitative real-time PCR (qRT-PCR). Gene expression was normalized to internal reference genes, and relative expression was calculated using the 2^^−ΔΔCt^ method. Data are presented as means ± standard deviation (n = 3). Asterisks indicate statistically significant differences between groups (**P < 0.01; ***P < 0.001; ****P < 0.0001; two-tailed Student’s t-test).

**Fig. S10**. KEGG pathway diagram of photosynthesis antenna proteins. RNA-seq–identified DEGs were mapped to the photosynthesis antenna proteins pathway (KEGG map00196). URed, green, and blue boxes represent upregulated, downregulated, and variably regulated genes, respectively. EC numbers are shown in boxes. Diagram was generated with KEGG Mapper.

**Fig. S111**. KEGG pathway diagram of Photosynthesis pathway. RNA-seq–identified DEGs were mapped to the photosynthesis antenna proteins pathway (KEGG map00195). Red, green, and blue boxes represent upregulated, downregulated, and variably regulated genes, respectively. EC numbers are shown in boxes. Diagram was generated with KEGG Mapper.

**Fig. S12**. KEGG pathway enrichment map of porphyrin metabolism. The porphyrin metabolism pathway (KEGG map00860) is shown with annotated enzymes and metabolites. Differentially expressed genes identified from RNA-seq analysis are mapped to the pathway. Red, green, and blue boxes represent upregulated, downregulated, and variably regulated genes, respectively. Enzyme EC numbers are shown in boxes. This visualization was generated using the KEGG Mapper tool.

**Fig. S13**. KEGG pathway diagram of Carbon fixation in photosynthetic organisms. RNA-seq–identified DEGs were mapped to the photosynthesis antenna proteins pathway (KEGG map00710). Red, green, and blue boxes represent upregulated, downregulated, and variably regulated genes, respectively. EC numbers are shown in boxes. Diagram was generated with KEGG Mapper.

**Fig. S14**. Venn diagram of gene expression between CK and CL groups. The Venn diagram illustrates the overlap of expressed genes between CK and CL samples. A total of 19803 genes were commonly expressed in both groups, while 58 and 55 genes were specifically expressed in CK and CL, respectively.

**Fig. S15**. Schematic representation of the synergistic regulation of carbon, nitrogen, and sulfur metabolism in photosynthesis. The diagram illustrates the interactions between the antenna protein pathway (ko000196), chlorophyll and porphyrin biosynthesis (ko00860), photosynthetic electron transport mechanism (ko00195), carbon fixation process (ko00710), and nitrogen metabolism, highlighting their regulatory roles in carbon, nitrogen, and sulfur metabolism. Arrows indicate the regulatory relationships between the metabolic pathways.

**Fig. S16**. The diagram illustrates the process of preparing LI-CDs via ultrasonic stirring and dialysis (top-left). LI-CDs enhance leaf growth, photosynthesis, and stress resistance, while soil factors influence microbial communities (bottom-left). The metabolic pathways related to photosynthesis and amino acid synthesis, including glutathione and methionine, are also shown (right).

**Table S1** 16S rRNA primers and quantitative real-time PCR

| Name | Sequence (5’ to 3’) |
| --- | --- |
| 338F | ACTCCTACGGGAGGCAGCAG |
| 806R | GGACTACHVGGGTWTCTAAT |
| IbACT-F | CTGGTGTTATGGTTGGGATGG |
| IbACT-R | GGG-GTGCCTCGGTAAGAAG |
| Ib4CL-F | AGCATTGAAGAAAGAGATAGTGATG |
| Ib4CL-R | GTTCTGTCTAACCATTGCCTTATC |
| IbHCT-F | CGCTTTTTACGCTTAGCAGCAA |
| IbHCT-R | TGGATTTCGGATGGGGGAAAG |
| IbCSE-F | GTACCACGTCTTCGCCGCCGA |
| IbCSE-R | CGCTCCTGTTCATGTACGGC |
| IbCOMT-F | TGCATTAAAAGATACAGTACTGG |
| IbCOMT-R | AACAAAGTATTTGTTAATTCCTGGT |
| IbANS-F | TGTGAACAGGGAGAAGGTGAG |
| IbANS-R | CATGATGAGTACGTCAGCGT |
| IbBZ1-F | GGGTCGGGCATAACGCCGCC |
| IbBZ1-R | TTGGAAGCCAAAAGGGCACC |

| Sample | CLY1 | CLY2 | CLY3 | CKY1 | CKY2 | CKY3 |
| --- | --- | --- | --- | --- | --- | --- |
| Raw reads | 47189414 | 44937438 | 44460482 | 55620010 | 47485846 | 46613468 |
| Raw bases | 7125601514 | 6785553138 | 6713532782 | 8398621510 | 7170362746 | 7038633668 |
| Clean reads | 46765690 | 44531550 | 44039216 | 55089156 | 47044510 | 46171044 |
| Clean bases | 7031391281 | 6693962842 | 6620985557 | 8277631336 | 7068918441 | 6935804099 |
| Error rate(%) | 0.012 | 0.012 | 0.0121 | 0.012 | 0.012 | 0.012 |
| Q20(%) | 98.7 | 98.72 | 98.68 | 98.7 | 98.72 | 98.7 |
| Q30(%) | 95.94 | 96.04 | 95.89 | 95.94 | 96.01 | 95.97 |
| GC content(%) | 44.73 | 44.28 | 44.59 | 44.84 | 44.77 | 45.1 |
| Total mapped | 42942488(91.82%) | 40838449(91.71%) | 40500915(91.97%) | 50541832(91.75%) | 43228801(91.89%) | 42394535(91.82%) |
| CDS | 50700072.0(82.23%) | 45839495.0(80.2%) | 47682528.0(82.23%) | 60857526.0(83.67%) | 51568510.0(83.36%) | 49079893.0(82.67%) |
| Intergenic | 2326962.0(3.77%) | 2299302.0(4.02%) | 1986818.0(3.43%) | 2137821.0(2.94%) | 1818294.0(2.94%) | 1782005.0(3.0%) |
| Introns | 3056428.0(4.96%) | 3441316.0(6.02%) | 3087807.0(5.32%) | 3263751.0(4.49%) | 2967810.0(4.8%) | 2769081.0(4.66%) |

**Table S2.** RNA-seq data quality and mapping statistics across all samples.

Note: Raw reads and clean reads indicate the number of reads before and after quality filtering, respectively. Q20 and Q30 represent the percentages of bases with Phred quality scores ≥20 and ≥30. GC content is the percentage of guanine and cytosine bases. "Total mapped" shows the number and percentage of reads successfully aligned to the reference genome. CDS, intergenic, and intron ratios indicate the distribution of reads across coding regions, non-coding regions, and introns.

**Table S3**. List of selected secondary metabolites significantly upregulated in vegetable sweetpotato leaves under LI-CDs treatment (CL vs. CK)

| Metabolite | (CL/CK)Regulate | Metabolite | (CL/CK)Regulate |
| --- | --- | --- | --- |
| Valylhydroxyproline | up | L-Glutamine | up |
| Maleamic Acid | up | N-Feruloyltyramine | up |
| Methionyl-Tyrosine | up | Tizanidine | up |
| Ginsenoside | up | Lichenin | up |
| Trilostane | up | Monocrotaline | up |
| Sulfamethizole | up | N-Acetyldopamine | up |
| L-Dopa | up | L-Serine | up |
| Norgalanthamine | up | N-Acetylproline | up |
| Glu Asn | up | Brassinolide | up |
| L-Asparagine | up | Prostaglandin J2 | up |
| Aspartyl-Cysteine | up | Carvone | up |
| Ustiloxin D | up | Traumatin | up |
| Prostaglandin A2 | up | Pipecolic Acid | up |
| Xanthine | up | Tyramine | up |
| 1-Methyladenine | up | Prostaglandin H2 | up |
| L-Histidine | up | Coniferin | up |
| Allantoic Acid | up | Albuterol | up |
| Caffeic Acid | up | Tryptamine | up |
| Indole-3-Carboxaldehyde | up |  |  |
